# Country-level gender inequality is associated with structural differences in the brains of women and men

**DOI:** 10.1073/pnas.2218782120

**Published:** 2023-05-08

**Authors:** André Zugman, Luz María Alliende, Vicente Medel, Richard A.I. Bethlehem, Jakob Seidlitz, Grace Ringlein, Celso Arango, Aurina Arnatkevičiūtė, Laila Asmal, Mark Bellgrove, Vivek Benegal, Miquel Bernardo, Pablo Billeke, Jorge Bosch-Bayard, Rodrigo Bressan, Geraldo F. Busatto, Mariana N. Castro, Tiffany Chaim-Avancini, Albert Compte, Monise Costanzi, Leticia Czepielewski, Paola Dazzan, Camilo de la Fuente-Sandoval, Marta Di Forti, Covadonga M. Díaz-Caneja, Ana María Díaz-Zuluaga, Stefan Du Plessis, Fabio L. S. Duran, Sol Fittipaldi, Alex Fornito, Nelson B. Freimer, Ary Gadelha, Clarissa S. Gama, Ranjini Garani, Clemente Garcia-Rizo, Cecilia Gonzalez Campo, Alfonso Gonzalez-Valderrama, Salvador Guinjoan, Bharath Holla, Agustín Ibañez, Daniza Ivanovic, Andrea Jackowski, Pablo Leon-Ortiz, Christine Lochner, Carlos López-Jaramillo, Hilmar Luckhoff, Raffael Massuda, Philip McGuire, Jun Miyata, Romina Mizrahi, Robin Murray, Aysegul Ozerdem, Pedro M. Pan, Mara Parellada, Lebogan Phahladira, Juan P. Ramirez-Mahaluf, Ramiro Reckziegel, Tiago Reis Marques, Francisco Reyes-Madrigal, Annerine Roos, Pedro Rosa, Giovanni Salum, Freda Scheffler, Gunter Schumann, Mauricio Serpa, Dan J. Stein, Angeles Tepper, Jeggan Tiego, Tsukasa Ueno, Juan Undurraga, Eduardo A. Undurraga, Pedro Valdes-Sosa, Isabel Valli, Mirta Villarreal, Toby T. Winton-Brown, Nefize Yalin, Francisco Zamorano, Marcus V. Zanetti, cVEDA, Anderson M. Winkler, Daniel S. Pine, Sara Evans-Lacko, Nicolas A. Crossley

**Affiliations:** ^a^Section on Development and Affective Neuroscience (SDAN), Emotion and Development Branch (E & D), National Institute of Mental Health, National Institutes of Health, Bethesda MD 20894; ^b^Department of Psychiatry, School of Medicine, Pontificia Universidad Católica de Chile, Santiago 8330077, Chile; ^c^Department of Psychology, Northwestern University, Evanston, IL 60208; ^d^Latin American Brain Health Institute (BrainLat), Universidad Adolfo Ibáñez, Santiago 7941169, Chile; ^e^Autism Research Centre, Department of Psychiatry, University of Cambridge, Cambridge CB2 8AH, United Kingdom; ^f^Department of Psychology, University of Cambridge, Cambridge CB2 3EB, United Kingdom; ^g^Department of Psychiatry, University of Pennsylvania, Philadelphia, PA 19104; ^h^Department of Child and Adolescent Psychiatry and Behavioral Science, The Children’s Hospital of Philadelphia, Philadelphia, PA 19104; ^i^Penn-Children’s Hospital of Philadelphia Lifespan Brain Institute, University of Pennsylvania, Philadelphia, PA 19104; ^j^Department of Child and Adolescent Psychiatry, Institute of Psychiatry and Mental Health, Hospital General Universitario Gregorio Marañón, Instituto de Investigación Sanitaria Gregorio Marañón (IISGM), Centro de Investigación Biomédica en Red Salud Mental (CIBERSAM), School of Medicine, Universidad Complutense, Madrid 28009, Spain; ^k^The Turner Institute for Brain and Mental Health, School of Psychological Sciences, Monash University, Melbourne, VIC 3168, Australia; ^l^Monash Biomedical Imaging, Monash University, Melbourne, VIC 3168, Australia; ^m^Department of Psychiatry, Faculty of Medicine and Health Sciences, Stellenbosch University, Cape Town 7602, South Africa; ^n^Centre for Addiction Medicine, National Institute of Mental Health and Neurosciences (NIMHANS), Bengaluru, Karnataka 560029, India; ^o^Barcelona Clinic Schizophrenia Unit, Hospital Clínic de Barcelona, Departament de Medicina, Institut de Neurociències (UBNeuro), Universitat de Barcelona (UB), Institut d’Investigacions Biomèdiques, August Pi i Sunyer (IDIBAPS), Centro de Investigación Biomédica en Red Salud Mental (CIBERSAM), Instituto de Salud Carlos III (ISCIII), Barcelona 08036, Spain; ^p^Laboratorio de Neurociencia Social y Neuromodulación, Centro de Investigación en Complejidad Social (neuroCICS), Facultad de Gobierno, Universidad del Desarrollo, Santiago 7610658, Chile; ^q^McGill Centre for Integrative Neuroscience, Ludmer Centre for Neuroinformatics and Mental Health, Montreal Neurological Institute, Montreal, QC H3A 2B4, Canada; ^r^McGill University, Montreal, QC H3A 2B4, Canada; ^s^Interdisciplinary Laboratory in Clinical Neuroscience (LiNC), Department of Psychiatry, Federal University of São Paulo, São Paulo 04039-032, Brazil; ^t^Departamento e Instituto de Psiquiatria, Hospital das Clínicas da Faculdade de Medicina da Universidade de São Paulo, São Paulo 05403-903, Brazil; ^u^Grupo de Investigación en Neurociencias Aplicadas a las Alteraciones de la Conducta (INAAC), Fleni-Consejo Nacional de Investigaciones Científicas y Técnicas Neurosciences Institute (INEU), Ciudad Autónoma de Buenos Aires C1428, Argentina; ^v^Department of Psychiatry and Mental Health, School of Medicine, University of Buenos Aires, Ciudad Autónoma de Buenos Aires C1114AAD, Argentina; ^w^Consejo Nacional de Investigaciones Científicas y Técnicas (CONICET), Ciudad Autónoma de Buenos Aires C1033AAJ, Argentina; ^x^Laboratory of Psychiatric Neuroimaging (LIM-21), Departamento e Instituto de Psiquiatria, Hospital das Clinicas, Faculdade de Medicina Universidade de São Paulo (HCFMUSP), Faculdade de Medicina Universidade de São Paulo, São Paulo SP 05403-903, Brazil; ^y^Institut d’Investigacions Biomèdiques August Pi i Sunyer (IDIBAPS), Barcelona 08036, Spain; ^z^Laboratory of Molecular Psychiatry, Centro de Pesquisa Clínica, Hospital de Clínicas de Porto Alegre, Porto Alegre RS 90035-007, Brazil; ^aa^Programa de Pós-Graduação em Psicologia, Instituto Psicologia, Universidade Federal do Rio Grande do Sul, Porto Alegre RS 90040-060, Brazil; ^bb^Department of Psychological Medicine, Institute of Psychiatry, Psychology and Neuroscience, King’s College London, London SE5 8AF, United Kingdom; ^cc^Laboratory of Experimental Psychiatry, Direction of Research, Instituto Nacional de Neurología y Neurocirugía, Mexico City 14269, Mexico; ^dd^Social, Genetic and Developmental Psychiatry Centre, Institute of Psychiatry, Psychology and Neuroscience, King’s College London, London SE5 8AF, United Kingdom; ^ee^National Institute for Health Research (NIHR), Maudsley Biomedical Research Centre, South London and Maudsley NHS Foundation Trust, King’s College London, London SE5 8AZ, United Kingdom; ^ff^Department of Psychiatry, Faculty of Medicine, University of Antioquia, Medellín 050011, Colombia; ^gg^Center for Neurobehavioral Genetics, Jane and Terry Semel Institute for Neuroscience and Human Behavior Los Angeles, University of California Los Angeles (UCLA), Los Angeles, CA 90024; ^hh^South African Medical Research Council (SA MRC), Genomics of Brain Disorders Unit, Cape Town 7505, South Africa; ^ii^Cognitive Neuroscience Center (CNC), Universidad de San Andres, Victoria, Ciudad Autónoma de Buenos Aires B1644BID, Argentina; ^jj^Global Brain Health Institute (GBHI), Trinity College Dublin (TCD), Dublin DO2 PN40, Ireland; ^kk^Global Brain Health Institute (GBHI), University of California San Francisco (UCSF), San Francisco, CA 94158; ^ll^Department of Psychiatry, Universidade Federal do Rio Grande do Sul (UFRGS), Hospital de Clinicas de Porto Alegre, Porto Alegre, RS 90035903, Brazil; ^mm^Integrated Program in Neuroscience, McGill University, Montreal, Quebec H3A 1A12B4 Canada; ^nn^Early Intervention Program, Instituto Psiquiátrico Dr. J. Horwitz Barak, Santiago 8431621, Chile; ^oo^School of Medicine, Universidad Finis Terrae, Santiago 7501015, Chile; ^pp^Laureate Institute for Brain Research, Tulsa, OK 74136; ^qq^Department of Integrative Medicine, NIMHANS, Bengaluru, Karnataka 560029, India; ^rr^Accelerator Program for Discovery in Brain disorders using Stem cells, Department of Psychiatry, NIMHANS, Bengaluru, Karnataka 560029, India; ^ss^Department of Psychiatry, Universidade Federal de São Paulo, São Paulo 04038-000, Brazil; ^tt^Department of Education, Information and Communications Technology (ICT) and Learning, Østfold University College, Halden 1757, Norway; ^uu^South African Medical Research Council (SA MRC) Unit on Risk and Resilience in Mental Disorders, Department of Psychiatry, Stellenbosch University, Stellenbosch 7505, South Africa; ^vv^Department of Psychiatry, Universidade Federal do Paraná (UFPR), Curitiba PR 80060-000, Brazil; ^ww^Department of Psychiatry, University of Oxford, Oxford OX3 7JX, United Kingdom; ^xx^Wellcome Centre for Integrative Neuroimaging (WIN), University of Oxford, Oxford OX3 9DU, United Kingdom; ^yy^NIHR Oxford Health Biomedical Research Centre, Oxford OX3 7JX, United Kingdom; ^zz^Oxford Health National Health Service (NHS), Foundation Trust, Oxford OX4 4XN, United Kingdom; ^aaa^Department of Psychiatry, Graduate School of Medicine, Kyoto University, Kyoto 606-8507, Japan; ^bbb^Clinical and Translational Sciences Lab, McGill University, Douglas Mental Health University Institute, Montreal, QC H4A 1R3, Canada; ^ccc^Department of Psychiatry, McGill University, Montreal, QC H3A 1A1, Canada; ^ddd^Department of Psychosis Studies, Institute of Psychiatry, Psychology and Neuroscience, King’s College London, London SE5 8AF, United Kingdom; ^eee^Department of Psychiatry and Psychology, Mayo Clinic, Rochester, Minnesota MN 55905; ^fff^National Institute of Developmental Psychiatry for Children and Adolescents, São Paulo 04038-000, Brazil; ^ggg^South African Medical Research Council (SA MRC) Unit on Risk and Resilience in Mental Disorders, Department of Psychiatry and Neuroscience Institute, University of Cape Town, Cape Town 7925, South Africa; ^hhh^Centre for Population Neuroscience and Stratified Medicine (PONS), Institute for Science and Technology for Brain-inspired Intelligence, Fudan University, Shanghai 200433, China; ^iii^PONS-Centre, Charité Mental Health, Dept of Psychiatry and Psychotherapy, Charité Campus Mitte, Berlin 10117, Germany; ^jjj^Integrated Clinical Education Center, Kyoto University Hospital, Kyoto 606-8397, Japan; ^kkk^Department of Neurology and Psychiatry, Faculty of Medicine, Clínica Alemana Universidad del Desarrollo Vitacura, Santiago 7650568, Chile; ^lll^Escuela de Gobierno, Pontificia Universidad Católica de Chile, Santiago 7820436, Chile; ^mmm^Research Center for Integrated Disaster Risk Management (CIGIDEN), Santiago 7820436, Chile; ^nnn^Canadian Institute for Advanced Research (CIFAR), Azrieli Global Scholars Program, CIFAR, Toronto, ON M5G 1M1, Canada; ^ooo^The Clinical Hospital of Chengdu Brain Science Institute, University of Electronic Science and Technology of China, Chengdu 610054, China; ^ppp^Centro de Neurociencias de Cuba, La Habana 11600, Cuba; ^qqq^Department of Physics, Universidad de Buenos Aires, Ciudad Autónoma de Buenos Aires C1428EGA, Argentina; ^rrr^Department of Neuroscience, Central Clinical School, Monash University, Melbourne, VIC 3004, Australia; ^sss^Department of Psychiatry, Alfred Health, Melbourne, VIC 3004, Australia; ^ttt^Centre for Affective Disorders, Department of Psychological Medicine, Institute of Psychiatry, Psychology and Neuroscience, King’s College London, London SE5 8AF, United Kingdom; ^uuu^South London and Maudsley National Health Service (NHS), Foundation Trust, London SE5 8AZ, United Kingdom; ^vvv^Unidad de Imágenes Cuantitativas Avanzadas, Departamento de Imágenes, Clínica Alemana de Santiago, Universidad del Desarrollo, Santiago 7650568, Chile; ^www^Facultad de Ciencias para el Cuidado de la Salud, Universidad San Sebastián, Santiago 7510602, Chile; ^xxx^Hospital Sírio-Libanês, São Paulo 01308-050, Brazil; ^yyy^Department of Human Genetics, University of Texas Rio Grande Valley, Brownsville, Texas TX 78520; ^zzz^Care Policy and Evaluation Centre, School of Economics and Political Science, London WC2A 2AE, United Kingdom

**Keywords:** gender inequality, structural brain MRI, sex differences

## Abstract

Gender inequality across the world has been associated with a higher risk to mental health problems and lower academic achievement in women compared to men. We also know that the brain is shaped by nurturing and adverse socio-environmental experiences. Therefore, unequal exposure to harsher conditions for women compared to men in gender-unequal countries might be reflected in differences in their brain structure, and this could be the neural mechanism partly explaining women’s worse outcomes in gender-unequal countries. We examined this through a random-effects meta-analysis on cortical thickness and surface area differences between adult healthy men and women, including a meta-regression in which country-level gender inequality acted as an explanatory variable for the observed differences. A total of 139 samples from 29 different countries, totaling 7,876 MRI scans, were included. Thickness of the right hemisphere, and particularly the right caudal anterior cingulate, right medial orbitofrontal, and left lateral occipital cortex, presented no differences or even thicker regional cortices in women compared to men in gender-equal countries, reversing to thinner cortices in countries with greater gender inequality. These results point to the potentially hazardous effect of gender inequality on women’s brains and provide initial evidence for neuroscience-informed policies for gender equality.

## Significance

Gender inequality is associated with worse mental health and academic achievement in women. Using a dataset of 7,876 MRI scans from healthy adults living in 29 different countries, we here show that gender inequality is associated with differences between the brains of men and women: cortical thickness of the right hemisphere, especially in limbic regions such as the right caudal anterior cingulate and right medial orbitofrontal, as well as the left lateral occipital, present thinner cortices in women compared to men only in gender-unequal countries. These results suggest a potential neural mechanism underlying the worse outcome of women in gender-unequal settings, as well as highlight the role of the environment in the brain differences between women and men.

Gender inequality profoundly impacts the society by creating an environment that significantly harms women. Women experience discrimination across many domains, including in education, the workplace, and in public office, and are disproportionately impacted by unpaid care work (1). However, gender inequality varies across countries as quantified using measures related to health, political representation, educational attainment, and labor market participation (2, 3). Such metrics have allowed to uncover country-level gender inequality associations with women’s worse mental health (4) and lower educational attainment (5).

Research on gender differences in brain structure could clarify possible reasons for gender differences in mental health problems (6). This would extend prior studies focused on endocrine or genetic contributions to gender differences in mental health problems (7, 8). Many studies find larger total intracranial volume in men, but other results are less consistent. This includes features of specific brain areas and findings for multiple morphometric properties such as thickness or surface area (9). Other work links brain structure to social and environmental factors. Such factors could differentially relate to brain structures across genders, contributing to inconsistency in studies of gender differences in brain structure. For example, exposure to early stimulation might increase gray matter cortical volumes in ways that persist in adulthood (10). Similarly, adverse childhood experiences could influence cortical surface area, thickness, and hippocampal volumes (11). Such adverse experiences might include exposure to hostile environments associated with stigma directed toward minority groups (12) or exposure to poverty (13). Leading hypotheses link these associations to stress physiology (14), accelerated aging process (15, 16), levels of environmental enrichment (17), and nutrition and health care (18). Women living in countries with high levels of gender inequality experience many of these same factors that are linked in prior research to brain structure. These adverse experiences include exposure to violence (19) as well as insufficient exposure to education and appropriate health care, both of which are considered indicators of gender inequality (2, 3). Therefore, it is also possible that brain structure is vulnerable to gender inequality: Women living in societies with high levels of gender inequality experience greater adversity, and this could negatively impact their brain development. Consistent with this perspective, a previous study based in 17 states in the United States found a trend-level association between hippocampal volume in 10-y-old girls and views on gender within the state (12). An international approach with increased variance in gender inequality could have more power to examine a potential effect. Gender inequality as an aggregate of these adverse factors, if presenting associations with brain structure, would link an important social determinant of health to the brain, which in turn might help explain gender-related differences in psychopathology (20). Such a study might also inform public policy in similar ways to studies examining other social correlates of brain structure (21).

To examine this possibility, differences in brain structure between healthy adult men and women from samples obtained in 29 countries were entered into a random-effects meta-analysis including a meta-regression, in which country-level gender inequality acted as an explanatory variable for the observed differences. Based on the findings from previous imaging studies on environmental factors, we explored associations with hemispheric and regional cortical thickness and surface area, as well as hippocampal volume, all measured using MRI. Cortical thickness and surface area have been widely used in previous multicenter studies (22). They are genetically and developmentally distinct (23, 24), and arguably provide a cleaner metric than volume, which is a composite of the two. Moreover, they relate in different ways to psychopathology across age groups and distinct forms of diagnosis (25, 26). For our metric of gender inequality, we combined the two most widely used national-level gender inequality metrics: the Gender Gap Index (2) and the Gender Inequality Index (3). We hypothesized that we would observe few structural differences in the brains of men and women in gender-equal countries, with differences appearing with higher levels of gender inequality.

## Results

**Fig. 1.**
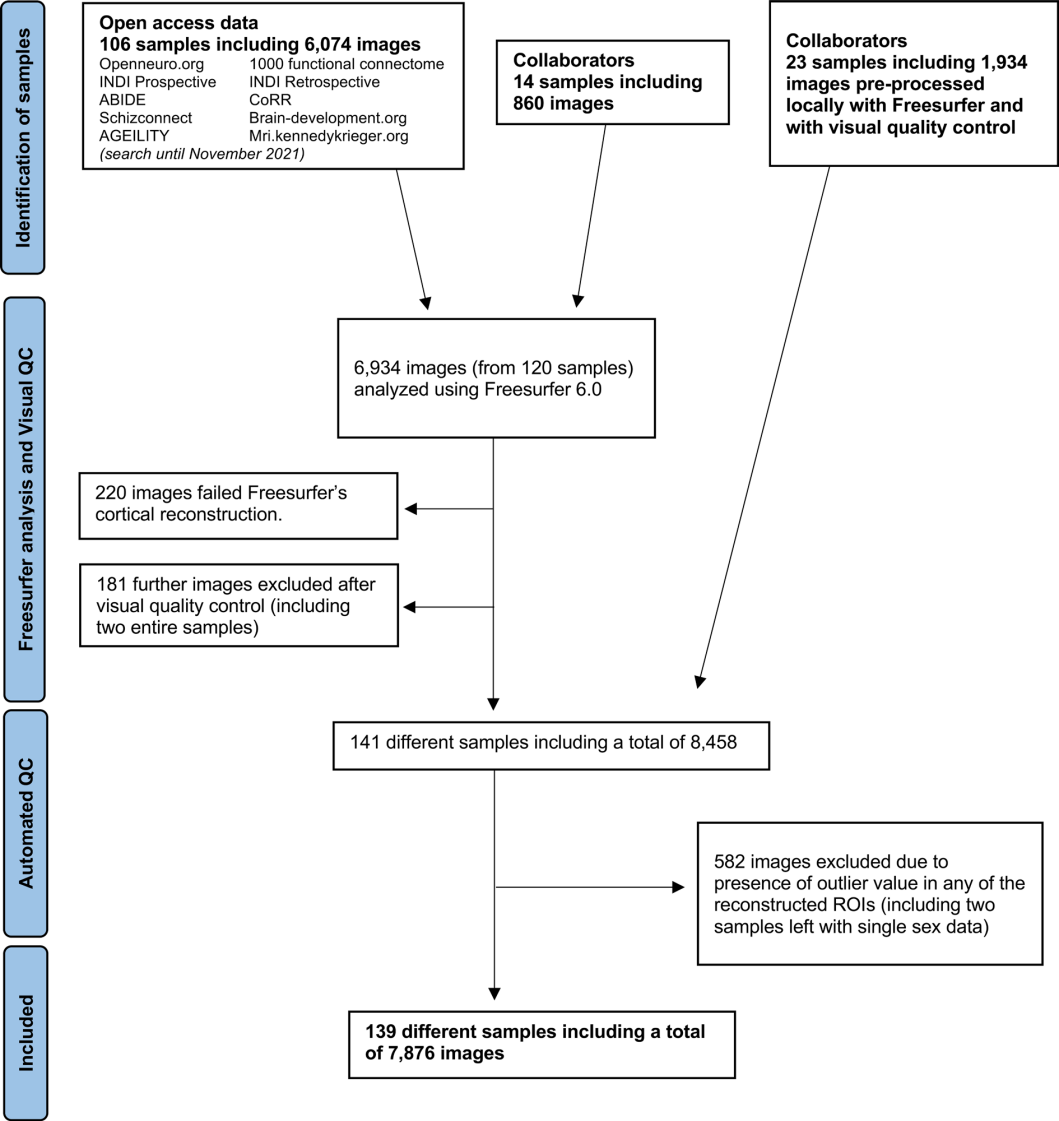
Flowchart of sample selection.

**Fig. 2.**
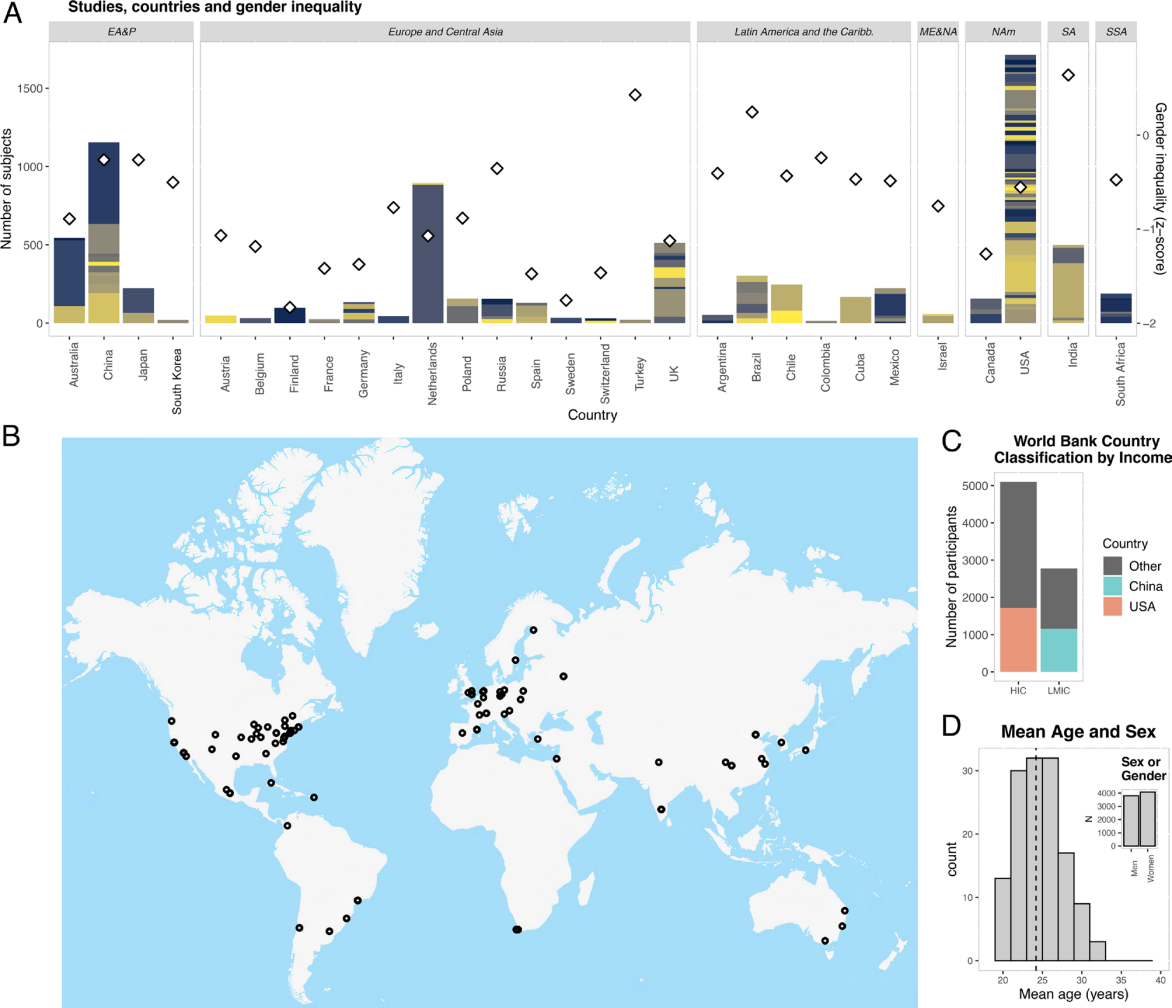
Demographic characteristics of samples included. (*A*) Number of participants included in each country (bars), with different colors denoting different studies/samples. The right *Y* axis and diamonds describe gender inequality Z-score, where higher values denote higher inequality. (*B*) Map showing the location of the main institutions that performed the studies included. (*C*) Number of participants from high-income countries (HIC) and low- and middle-income countries (LMIC), highlighting participants from China and the United States. (*D*) Histogram with mean age and sex within and across the samples, respectively. EA&P = East Asia and Pacific; ME&NA = Middle East and North Africa; NAm = North America; SA = South Asia; SSA = Sub-Saharan Africa.

This study included 139 samples from 29 different countries, totaling 7,876 MRI scans from 4,078 women and 3,798 men (Figs. 1 and 2 and Dataset S1). Nearly 35.26% of the participants lived in low- and middle-income countries. The median mean age across samples was 24.19 y (range 18.83 to 31.69 y). To account for a potential effect of age within samples, we regressed the linear effect of age in each sample.

**Fig. 3.**
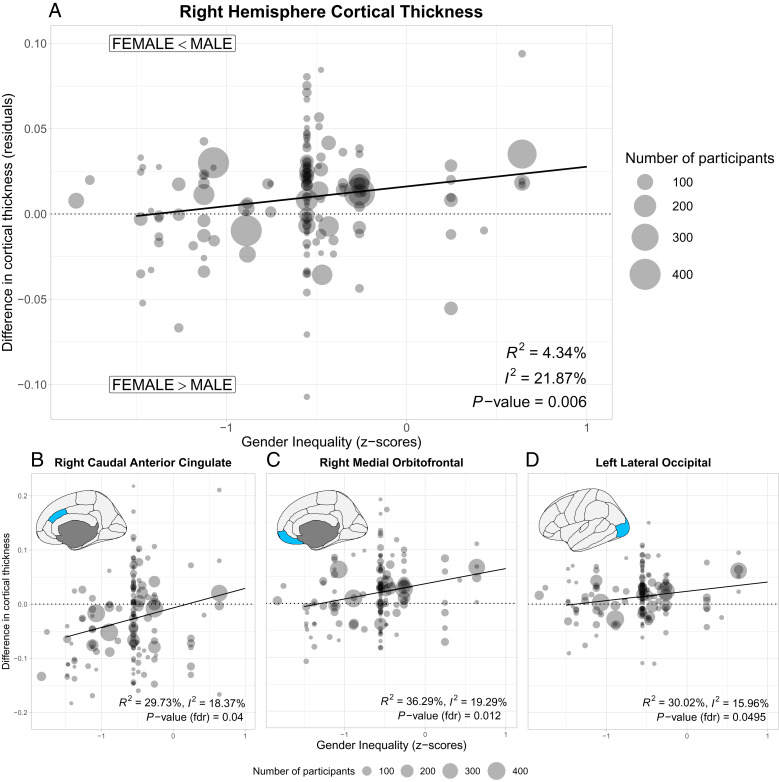
Associations between country-level gender inequality and the average difference of the cortical thickness between women and men. (*A*) Right hemisphere. Circles represent the thickness difference between men and women in a specific sample; their size represents the number of participants. Negative values of the gender inequality index describe a higher equality between men and women. Solid line represents fit of main analysis. (*B*–*D*) Significant associations between gender inequality and regional cortical thickness after controlling for multiple comparisons.

Analyses of hemisphere-wide average cortical thickness revealed a significant association with gender inequality in the right (Fig. 3*A*, beta 0.012 (95% CI 0.0033 to 0.020), *P* = 0.006, with an *R*^2^ = 4.34%), but not left hemisphere (beta 0.008 (95% CI −0.001 to 0.0155), *P* = 0.07; *SI Appendix*, Fig. S1). Countries with greater gender equality showed practically no differences in cortical thickness in the right hemisphere between the sexes. Sex differences emerged in countries with greater gender inequality, with men having thicker cortices than women. Reliability analyses showed that this result was not driven by a single sample (*SI Appendix*, Fig. S2). National gender inequality indices are associated with economic development (27). Our result remained significant after controlling for the logarithm of the per capita gross domestic product [beta 0.015 (95% CI 0.004 to 0.026), *P* = 0.0065]. Analyses looking at the association between cortical thickness with gender inequality in women only found that cortical thickness in the right hemisphere tended to decrease with higher gender inequality, albeit not significantly [*SI Appendix*, Fig. S3, beta −0.022 (95% CI −0.047 to 0.0036), *P* = 0.093]. We found no evidence of an association in men [beta −0.0075 (95% CI −0.036 to 0.021), *P* = 0.6].

Analyses on brain regions of interest (68 subregions tested) showed that cortical thickness differences in three brain regions correlated significantly with gender inequality after correcting for multiple testing using false-discovery rate: right caudal anterior cingulate gyrus [beta 0.036 (95% CI 0.014 to 0.058), *P_FDR_* = 0.040], right orbitofrontal gyrus [beta 0.028 (95% CI 0.014 to 0.043), *P_FDR_* = 0.012], and left lateral occipital cortex [beta 0.017 (95% CI 0.006 to 0.028), *P_FDR_* = 0.0495; Fig. 3 *B*–*D*]. These three areas shared the same pattern: There were no differences (or even thicker regional cortices in the case of the caudal anterior cingulate gyrus) in women in countries with greater gender equality, reversing to thinner cortices in countries with greater gender inequality. The association with the right caudal anterior cingulate gyrus also remained significant after controlling for economic development [beta 0.055 (95% CI 0.027 to 0.084), *P_FDR_* = 0.0098], but was no longer significant for the right medial orbitofrontal gyrus (*P_FDR_* = 0.064) or the left lateral occipital cortex (*P_FDR_* = 0.13).

Further analyses demonstrated that our cortical thickness results were consistent across several methodological variations. One could hypothesize that the association was driven by samples from China and the United States, the two countries that contributed the greatest number of images. Our results were not modified substantially when we excluded studies from those two countries (*SI Appendix*, Fig. S4). The results were also consistent when considering the individual noncombined inequality indices (*SI Appendix*, Fig. S5), including only studies performed on 3T MRI scanners (*SI Appendix*, Fig. S6), excluding very small studies (*SI Appendix*, Fig. S7), or exclusively analyzing those with visual quality checks performed by the same two researchers (*SI Appendix*, Fig. S8).

There were no significant associations between gender inequality and hemispheric or regional surface area (*SI Appendix*, Fig. S9), hippocampal volumes (*SI Appendix*, Fig. S10), or with total intracranial volume (*SI Appendix*, Fig. S11).

## Discussion

The results show that country-level gender inequality is related to the average structural brain differences between women and men in cortical thickness. The effect seen was a global one, significant in the cortical thickness of the right hemisphere.

Gender inequality indices are composite measures that incorporate diverse experiences that might be mediating their effect on the brain through different biological mechanisms. However, we could hypothesize about the predominant underlying mechanisms based on the localized brain regions in which a significant association was found, namely, the anterior cingulate gyrus and orbitofrontal gyrus. These regions have been related to several aspects of emotional control, including resilience to adversity (28), responses to inequity (29), or negative social comparisons (30). Changes in these regions have also been found in pathological conditions where stress is considered a central mechanism, including thinning in depression (25), or reduced volume in posttraumatic stress disorder (31). Stress would lead to these macroscopic changes through dendritic remodeling and synaptic pruning, possibly mediated by stress hormones (32). Overall, the observed association may result from exposure to an adverse environment and subsequent stress response throughout life. This would imply that sex differences in the thickness of those regions would be smaller in early development and increase during aging. This resonates with evidence highlighting the role of gender inequality in the higher prevalence of depression in girls which appears in adolescence (33). Other data support this hypothesis about the timing of the observed changes. For example, stress in the adult brain seems to correlate most consistently with cortical thickness rather than cortical surface area (34, 35); similarly, hippocampal volume relates most consistently to early life stress (32). However, other mechanisms could contribute to such changes. Women could have lower access to beneficial, enriched environments, which could alter their brain structure through higher dendritic branching and increased synapse formation (17). Indeed, the composite indices of gender inequality incorporate the lower educational opportunities of women compared to men. The observed associations could also relate to very early disturbances in development, particularly since cortical thickness peaks early in brain maturation (23). Our study could not examine further which of these mechanisms were involved, since many types of adverse experiences coexist across societies (36). New studies looking at specific populations in which they are not as correlated might inform about the underlying mechanisms. Further insights could come from studies examining differences between cohorts that have been exposed to changing levels of adversity over time, particularly since some domains might improve earlier or be subject to specific policy interventions (such as improving perinatal care). A longitudinal temporal view would also strengthen the case for a causality mechanism in the observed association.

While analyses performed on small, nonrandom samples may not be representative of the population, we included multiple studies and from different cities in each country when possible, to increase representativeness. Neuroimaging is still an expensive tool, and despite our data including more than a third of participants from low- and middle-income economies, only India represented the low- and lower-middle-income groups. By focusing on the difference between the brains of women and men within each site included, our analyses were less likely to be biased by factors related to the MRI scanner and sequence used (37), or the ethnic and socioeconomic background of the population studied (13, 38).

These results highlight the relevance of the macrosocial environment where sex differences in brain structure are manifested. Future studies will need to examine the mechanisms involved, their moderating factors, and their timing, providing new opportunities for neuroscience-informed policies (21) to promote gender equality.

## Materials and Methods

We included samples that reported structural MRI data (T1 weighted) acquired on 1.5T and 3T scanners from healthy adults aged 18 to 40 y (inclusive). Although “gender” is related to the individual expression of identity, gender inequality measured across countries is usually reduced to biological sex, which is also collected in many research data. We therefore use the term sex of participants, acknowledging incomplete overlap with gender identity. Data were obtained both from open-access platforms and collaborators across the world (Fig. 1), and they all had local institutional ethical approval. Images were analyzed with FreeSurfer, focusing on cortical thickness and surface area from 68 regions of the Desikan–Killiany’s template and the two hemispheres, as well as the hippocampal volumes. Age was linearly regressed out from each sample (23). Following previous studies focusing on sex differences beyond brain size (9), total intracranial volume was also controlled for in the surface area and hippocampal volume analyses (*SI Appendix*, Fig. S12). When examining localized (regional) associations, we corrected results for multiple testing using false discovery rate (FDR). Further details of the methods can be found in *SI Appendix*. Group-level data and the script with the main analyses can be downloaded from https://github.com/zugmana/CLGI. Dataset S1 provides detailed information on how to gain access to individual-subject data from the different sites included.

## Supplementary Material

Appendix 01 (PDF)

Dataset S01 (XLSX)

Dataset S02 (DOCX)

## Data Availability

Group data have been deposited in Github (https://github.com/zugmana/CLGI) (39). Individual participant data can be accessed from different sources as detailed in Dataset S1. Some datasets require consent from principal investigators named there.
