# Supplementary material for: Country-level gender inequality is associated with structural differences in the brains of women and men
Source: Proc Natl Acad Sci U S A. 2023 May 8;120(20):e2218782120. doi: 10.1073/pnas.2218782120 (PMC10193926; doi:10.1073/pnas.2218782120)
Supplement: Supplementary file 1 — Appendix 01 (PDF) [file pnas.2218782120.sapp.pdf]

PNAS

Supplementary Information for

Country-level gender inequality is associated with structural differences in the brains of women and men

André Zugman, Luz María Alliende, Vicente Medel, Richard Bethlehem, Jakob Seidlitz, Grace Ringlein, Celso Arango, Aurina Arnatkevičiūtė, Laila Asmal, Mark Bellgrove, Vivek Benegal, Miquel Bernardo, Pablo Billeke, Jorge Bosch-Bayard, Rodrigo Bressan, Geraldo F. Busatto, Mariana N. Castro, Tiffany Chaim-Avancini, Albert Compte, Monise Costanzi, Leticia Czepielewski, Paola Dazzan, Camilo de la Fuente-Sandoval, Marta Di Forti, Covadonga M. Díaz-Caneja, Ana María Diaz-Zuluaga, Stefan Du Plessis, Fabio LS Duran, Sol Fittipaldi, Alex Fornito, Nelson B Freimer, Ary Gadelha, Clarissa Gama, Ranjini Garani, Clemente Garcia-Rizo, Cecilia Gonzalez Campo, Alfonso Gonzalez-Valderrama, Salvador Guinjoan, Bharath Holla, Agustín Ibañez, Daniza Ivanovic, Andrea Jackowski, Pablo Leon-Ortiz, Christine Lochner, Carlos López-Jaramillo, Hilmar Luckhoff, Raffael Massuda, Philip McGuire, Jun Miyata, Romina Mizrahi, Robin Murray, Aysegul Ozerdem, Pedro Pan, Mara Parellada, Lebogang Phahladira, Juan P Ramirez-Mahaluf, Ramiro Reckziegel, Tiago Reis Marques, Francisco Reyes-Madrigal, Annerine Roos, Pedro Rosa, Giovanni Salum, Freda Scheffler, Gunter Schumann, Mauricio Serpa, Dan J Stein, Angeles Tepper, Jeggan Tiego, Tsukasa Ueno, Juan Undurraga, Eduardo A. Undurraga, Pedro Valdes-Sosa, Isabel Valli, Mirta Villarreal, Tobias T Winton-Brown, Nefize Yalin, Francisco Zamorano, Marcus V Zanetti, cVEDA, Anderson M. Winkler, Daniel S. Pine, Sara Evans-Lacko and Nicolas A. Crossley*.

* Corresponding author: Nicolas A. Crossley

Email: ncrossley@uc.cl

This file includes:

Extended Methods
Extended List of Acknowledgements
Figures S1 to S12
cVEDA author list

Extended Methods

Information sources and search strategy
We were particularly interested in including participants studied in MRI scanners across many countries, since our study examined the between-country variance. We accomplished this through two methods:
- We included open-access data reporting MRI images from healthy adults within the age range of interest, collating several databases until November 2021 (see Figure 1). We made the effort to include all pertinent data, particularly from as many countries as possible, but trying to avoid that our final sample was dominated by countries over-represented in the scientific literature, such as the USA or China. The reasoning behind this was to avoid the meta-regression results being solely driven by differences between these few countries, possibly by confounders. This particularly applied to one specific database (openneuro.org) in which many studies came from the most represented country, USA. In that case, we finally included 29% of eligible data from this country. Had we included all the data in this database from the USA, it would have doubled its representation in the total sample in our study from 22% to 43%. To check if such a convenience sample of neuroimaging studies (that are also reporting results from convenience samples) could bias our analyses, we performed confirmatory analyses excluding all studies from the USA and China.
- We included data from collaborators across the world.

Eligibility criteria
We included samples that reported structural MRI data (T1 weighted) from healthy adults aged 18 to 40 years (inclusive). This age range was selected since it is a period when development and aging processes are less marked. We excluded participants with known mental health or neurological disorders. However, we did not define specific assessments that needed to be performed in each site to define a participant as healthy, but relied on the site definition. To be considered initially for analysis, samples had to include men and women and at least 15 participants, as well as being approved by their local ethics committee. The final number of participants could have been lower if participants were excluded during later quality control checks. Although “gender” is related to the individual expression of identity, gender inequality measured across countries is usually reduced to biological sex. This dichotomous definition is also collected in many research data. We therefore use the term sex of participants, acknowledging incomplete overlap with gender identity.

We focused on images acquired on 1.5T and 3T MRI scanners. We excluded samples from 7T MRI scanners since they require modifications to the Freesurfer processing pipeline used and their results might not be entirely comparable (1). High-field (7T) MRI scanners are also more likely to be in high-income countries, potentially introducing a bias, particularly considering the association between gender inequality and economic development discussed later. We also explored the potential effect of the different types of scanners by analyzing only studies done on a 3T MRI scanner.

Preprocessing of imaging data
All imaging data were processed using FreeSurfer’s cortical reconstruction pipeline recon-all (see Dataset S1 for details of the specific version used). In line with other studies using multi-center data (2), we used Desikan-Killiany’s cortical parcellation (3), including cortical thickness and separately surface area of 68 cortical regions of interest (ROI) and the two hemispheres.

Quality control of the process was based on an initial visual quality control. As shown in Dataset S1, this was performed locally by some collaborating groups, and the rest performed by two reviewers (AZ and NAC). To check whether this introduced any bias, we performed an analysis only including studies which were quality controlled by the two reviewers mentioned above. It was then followed up by an automatic quality control where subjects were excluded if any of the ROIs, either in thickness or surface area, were outliers defined by Tukey’s fence (4):

[1] $[Q_1-k(Q_3-Q_1), Q_3+k(Q_3-Q_1)]$

Where *Q* refers to the respective quartile and a value of *k*=3 was used. We also examined a scatterplot of the difference between women and men in intracranial volume, excluding one extreme outlier.

Following our previous work (5), age was linearly regressed out from each sample and residuals of cortical thickness and surface area were used in subsequent analyses.

We were particularly interested in sex differences that were not due to head size (6). We therefore included total intracranial volume as a confounder in the surface area analysis, with secondary analyses using total brain surface. We did not correct for head size in the thickness analysis as suggested in the literature (7). Our data confirmed that there is little association between thickness and total intracranial volume (Figure S7).

Country-level measures of gender inequality and economic development

We used a combination of the two most-known gender inequality indices: the United Nations' Gender Inequality Index, and the World Economic Forum's Global Gender Gap Index. To combine both tests, we first *z*-normalized them including all countries in the world for which these indices are available; we then inverted the sign of the World Economic Forum's normalized index, and took the mean of these metrics. We used a single time point of these metrics, namely 2019, before the start of the pandemic as indices could have changed substantially.

Economic development was indexed using the per capita Gross Domestic Product (GDP) of the country as collated by the World Economic Forum and published for 2019.

Meta-regression analyses

Differences in brain structure between men and women were entered into a random-effects meta-analysis including a meta-regression, in which country-level gender inequality acted as an explanatory variable for the observed differences between countries. We performed this analysis independently for the two hemispheric values of cortical thickness and surface area, and for 68 regions of interest from the Desikan-Killiany atlas. We used a random-effects model with weights based on the inverse of the variance of the imaging metric examined, modeling the between-study variance using the Paule and Mandel estimator (8). When examining localized (regional) associations, we corrected results for multiple testing using false-discovery-rate (FDR) (9).

As described in the literature (13), per capita GDP was correlated with our summary measure of Gender Inequality (R=-0.43, P-value<0.0001). We therefore also performed analyses including it as an extra moderator.

All statistical analyses were performed in R (4.02) using the *metafor* package (14).

Sensitivity analyses

To ensure further that results were not driven by any single site, we additionally performed a jackknife analysis (leave one out analysis). As a reliability analysis, we also report separately the association between the brain metrics and the World Economic Forum and United Nations gender inequality indicators.

References

1. F. Lüsebrink, A. Wollrab, O. Speck, Cortical thickness determination of the human brain using high resolution 3 T and 7 T MRI data. *Neuroimage* **70**, 122–131 (2013).

2. P. M. Thompson, *et al.*, The ENIGMA Consortium: Large-scale collaborative analyses of neuroimaging and genetic data. *Brain Imaging Behav.* **8**, 153–182 (2014).

3. R. S. Desikan, *et al.*, An automated labeling system for subdividing the human cerebral cortex on MRI scans into gyral based regions of interest. *Neuroimage* **31**, 968–980 (2006).

4. J. W. Tukey, *Exploratory data analysis* (Reading, MA, 1977).

5. R. A. I. Bethlehem, *et al.*, Brain charts for the human lifespan. *Nature*, 1–11 (2022).

6. L. Eliot, A. Ahmed, H. Khan, J. Patel, Dump the “dimorphism”: Comprehensive synthesis of human brain studies reveals few male-female differences beyond size. *Neurosci. Biobehav. Rev.* **125**, 667–697 (2021).

7. eTIV - estimated Total Intracranial Volume, aka ICV. *FreeSurferWiki*.

8. A. A. Veroniki, *et al.*, Methods to estimate the between-study variance and its uncertainty in meta-analysis. *Res. Synth. Methods* **7**, 55–79 (2016).

9. Y. Benjamini, Y. Hochberg, Controlling the false discovery rate: a practical and powerful approach to multiple testing. *J. R. Stat. Soc. Ser. B* **57**, 289–300 (1995).

10. H. G. Schnack, *et al.*, Mapping reliability in multicenter MRI: Voxel-based morphometry and cortical thickness. *Hum. Brain Mapp.* **31**, 1967–1982 (2010).

11. K. G. Noble, *et al.*, Family income, parental education and brain structure in children and adolescents. *Nat. Neurosci.* **18**, 773–778 (2015).

12. B. Holla, *et al.*, A series of five population-specific Indian brain templates and atlases spanning ages 6–60 years. *Hum. Brain Mapp.* **41**, 5164–5175 (2020).

13. S. Jayachandran, The roots of gender inequality in developing countries. *economics* **7**, 63–88 (2015).

14. W. Viechtbauer, Conducting meta-analyses in R with the metafor package. *J. Stat. Softw.* **36**, 1–48 (2010).

Extended list of acknowledgements

NAC, JU and AG-V are supported by ANID-PIA-ACT192064, ANID-FONDECYT Regular 1180358 and 1200601. AZ, DSP and AMW are supported by the by the Intramural Research Program of the NIMH through ZIA-MH002781 and ZIA-MH002782. MB is supported by the Instituto de Salud Carlos III, the Spanish Ministry of Science, Innovation and Universities, the European Regional Development Fund (ERDF/FEDER)(PI08/0208, PI11/00325, PI14/00612); CIBERSAM; CERCA Program; Catalan Government, the Secretariat of Universities and Research of the Department of Enterprise and Knowledge (2017SGR1355) and Institut de Neurociències, Universitat de Barcelona. GFB is supported by FAPESP grant number 14/50873-3. CDC has received grant support from Instituto de Salud Carlos III (PI17/00481, JR19/00024, PI20/00721). AI is partially supported by grants of Takeda CW2680521; CONICET; FONCYT- PICT (2017-1818, 2017-1820); ANID-FONDECYT Regular (1210195, 1210176, 1220995); ANID-FONDAP (15150012); ANID-FONDEFID (20I10152 and ID22I10029), ANID-PIA-Anillo (ACT210096); and the Multi-Partner Consortium to Expand Dementia Research in Latin America (ReDLat), funded by the National Institutes of Aging of the National Institutes of Health under award number R01AG057234, an Alzheimer's Association grant (SG-20-725707-ReDLat), the Rainwater Foundation, and the Global Brain Health Institute (GBHI). CGR is supported by project PI20/00661 from Instituto de Salud Carlos III and co-financed by the European Union (Feder) "a way of making Europe". GS is supported by the Horizon 2020 funded ERC Advanced Grant 'STRATIFY' (695313), RFIS-NSFC grant (82150710554) and the DFG FKZ 458317126. JM and TU are supported by the Japan Society for the Promotion of Science and the Ministry of Education, Culture, Sports, Science and Technology (KAKENHI) grant numbers 26461767, 17H04248, 18H05130 and 20H05064, and the Japan Agency for Medical Research and Development grant numbers JP18dm0307008 and JP21uk1024002. EU was supported by ANID-FONDAP (1522A0005) and The Canadian Institute for Advanced Research CIFAR (Humans and the Microbiome).

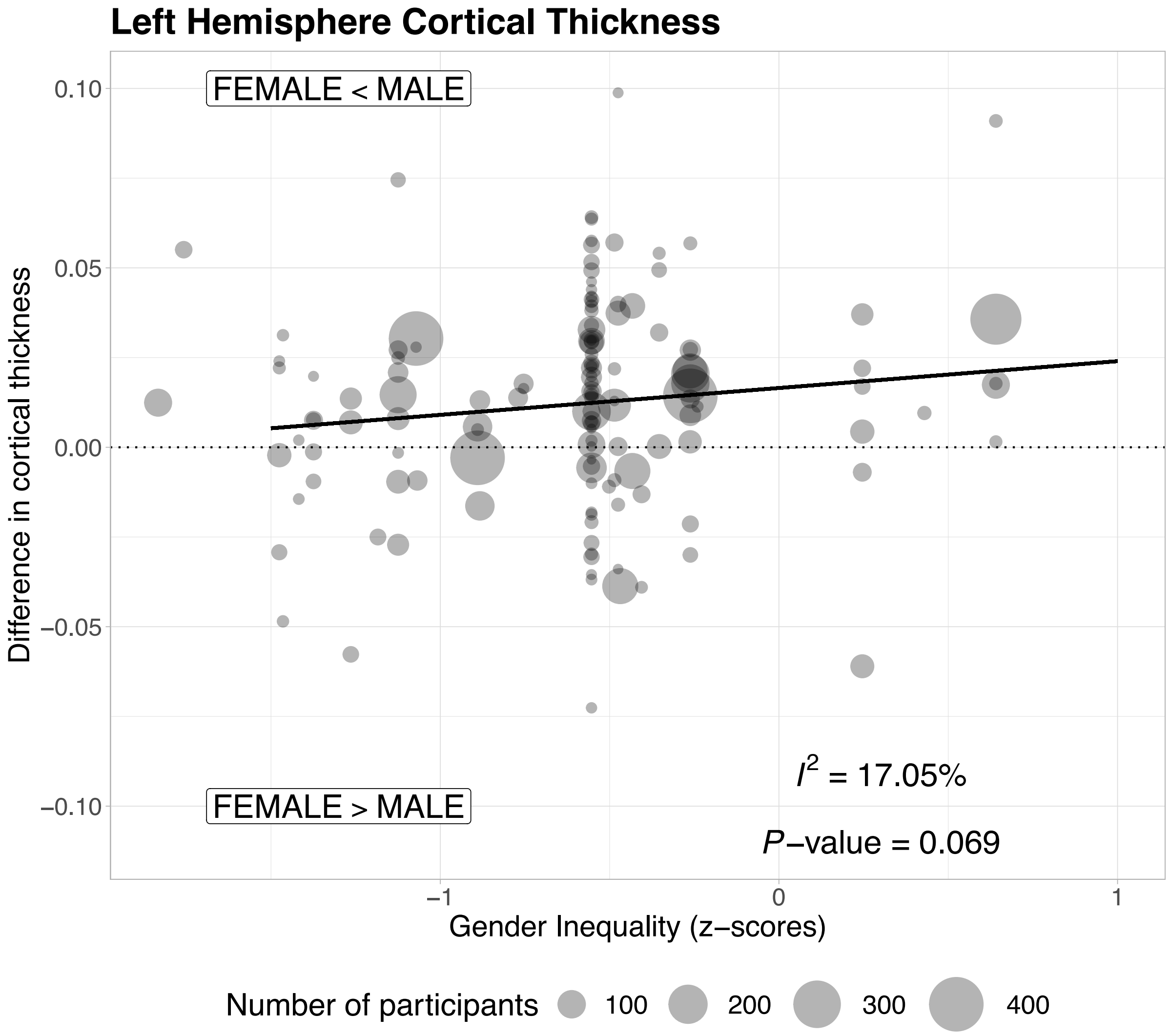

Figure S1. Association between gender inequality and mean left hemisphere cortical thickness. The beta associated with inequality was 0.0075 (95%CI -0.0006 to 0.016). When controlling for the log(per capita GDP), this association was significant, with a beta of 0.0113 (95%CI 0.0006 to 0.0219) and a P-value of 0.038.

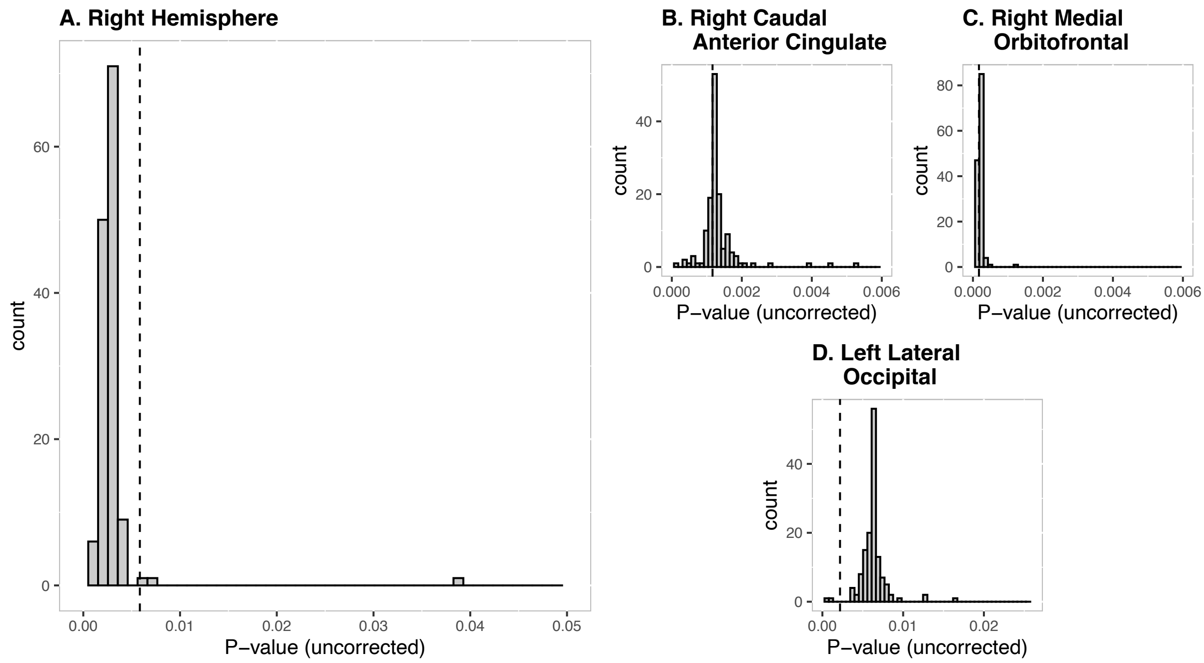

Figure S2. Reliability analyses of cortical thickness findings. Jackknife analyses shown, with dashed lines representing statistical significance found in the whole sample analyses. Note that the mean right hemisphere thickness, as well as right caudal anterior cingulate and right medial orbitofrontal findings are consistent. The left lateral occipital finding is more dependent on the specific sample included.

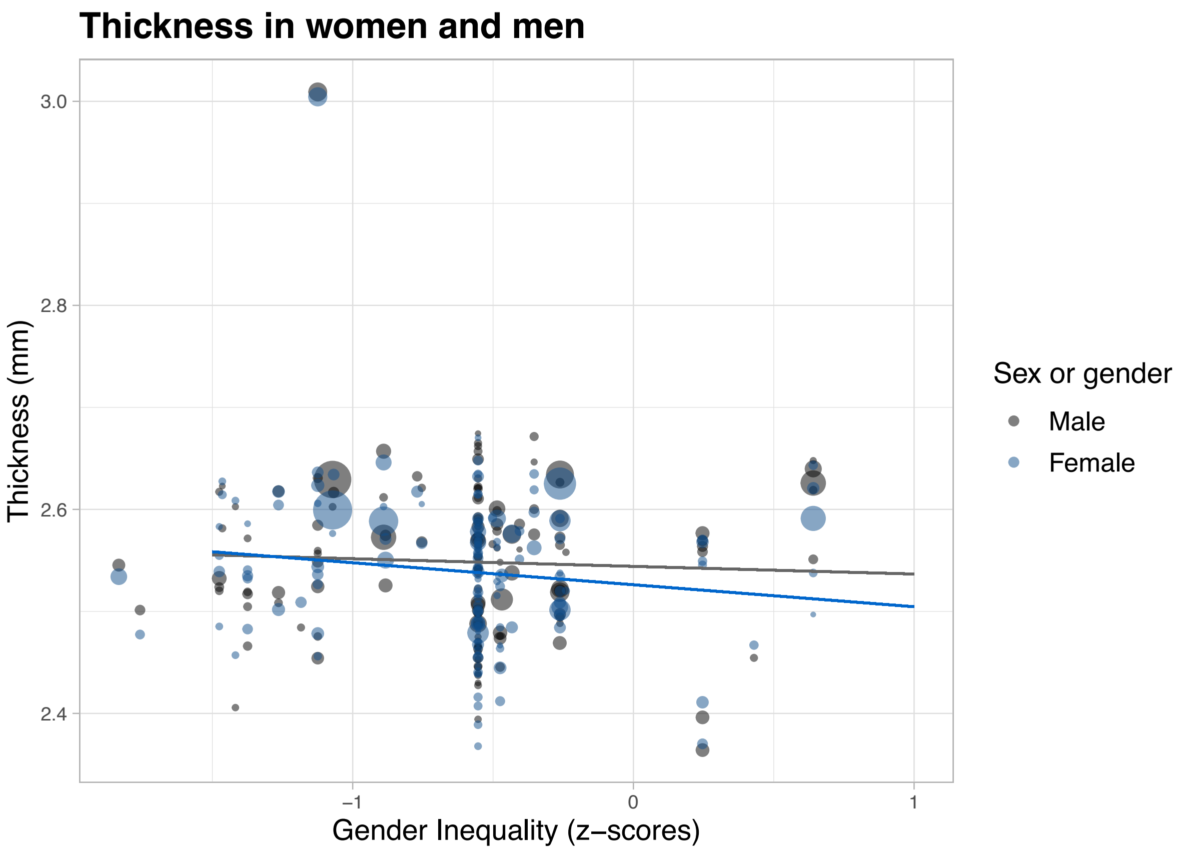

Figure S3. Association between gender inequality and women's and men's right hemisphere cortical thickness. Beta of association in women was -0.022 (95%CI -0.047 to 0.0036, *P* = 0.093) and for men a beta of -0.008 (95%CI -0.036 to 0.021, *P* = 0.6).

Differences in cortical thickness excluding China and USA

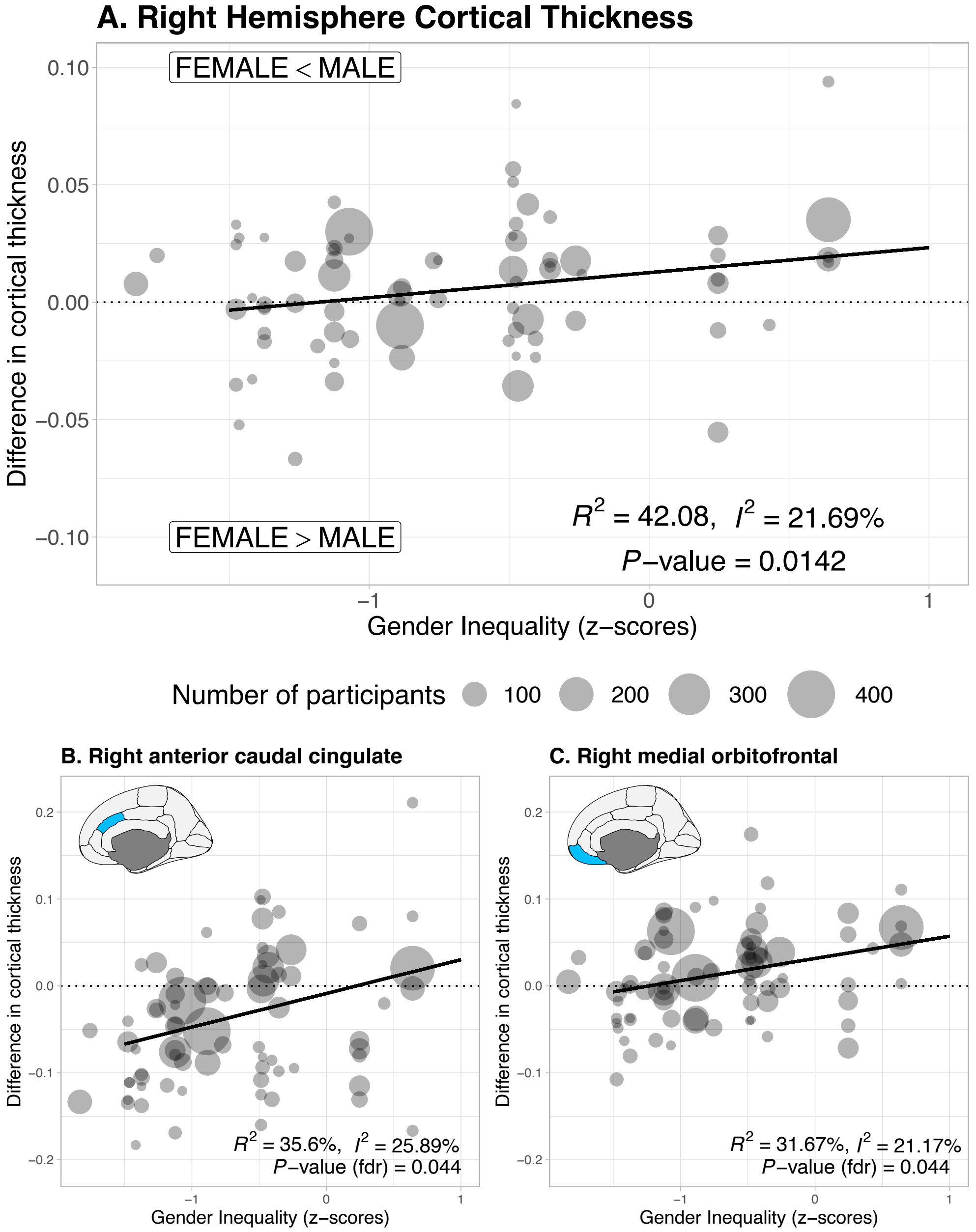

Figure S4. Analyses of cortical thickness excluding studies from China and the USA. USA and China were the countries that contributed the largest number of participants (22% and 15% of the total sample respectively). This large proportion already considered a convenience sampling approach that did not include all open access data from these countries. This raised the possibility that the findings were mostly driven by the differences between these two countries. We included 75 samples with a total of 5,008 participants. The results were consistent with the main analyses (compare to Figure 3). Only the lateral occipital cortex was no longer significant in this analysis, after controlling for multiple comparisons ($P = 0.012$, $P_{FDR} = 0.13$).

Thickness right hemisphere

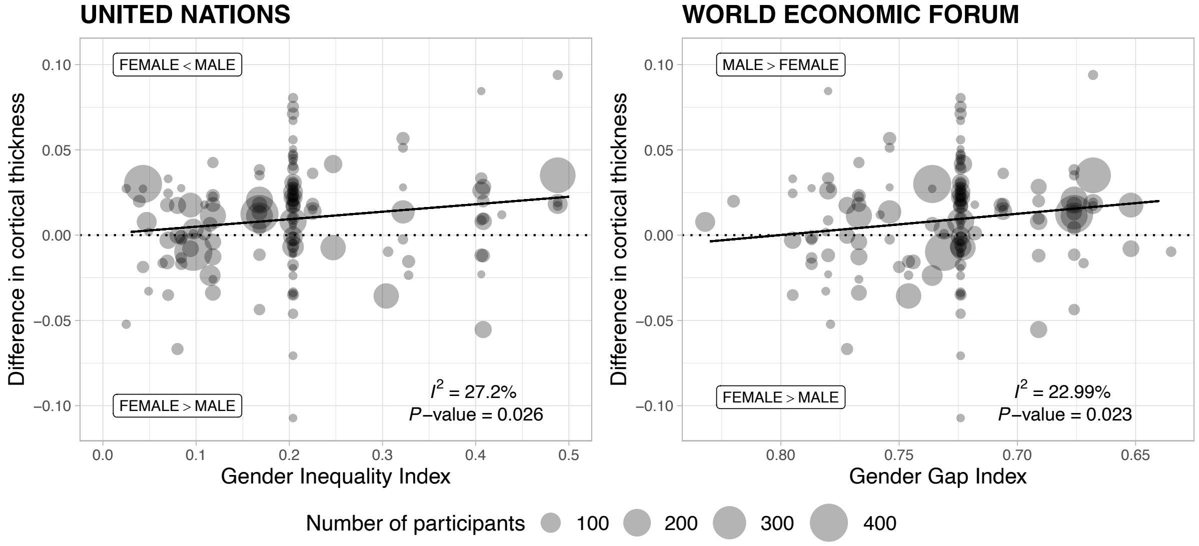

	Uncorrected P-values		
	Combined Index	*World Economic Forum*	*United Nations*
**Right hemisphere**	0.006	0.023	0.026
**Right caudal anterior cingulate**	0.0012	0.007	0.015
**Right medial orbitofrontal**	0.0002	0.001	0.005
**Left lateral occipital**	0.0022	0.041	0.005

Figure S5. Thickness analyses and the UNDP's gender inequality index or the World Economic Forum's gender gap report. (A) Results for the mean right hemisphere. The x-axis on the World Economic Forum indicator has been reversed so that it can be read congruently with the UNDP indicator (left side of x-axis (lower UN Gender Inequality Index and higher World Economic Forum's Gender Gap Index): more gender-equal countries). (B) Uncorrected P-values for the identified regions in the main analyses.

Differences in cortical thickness in 3T MRI studies

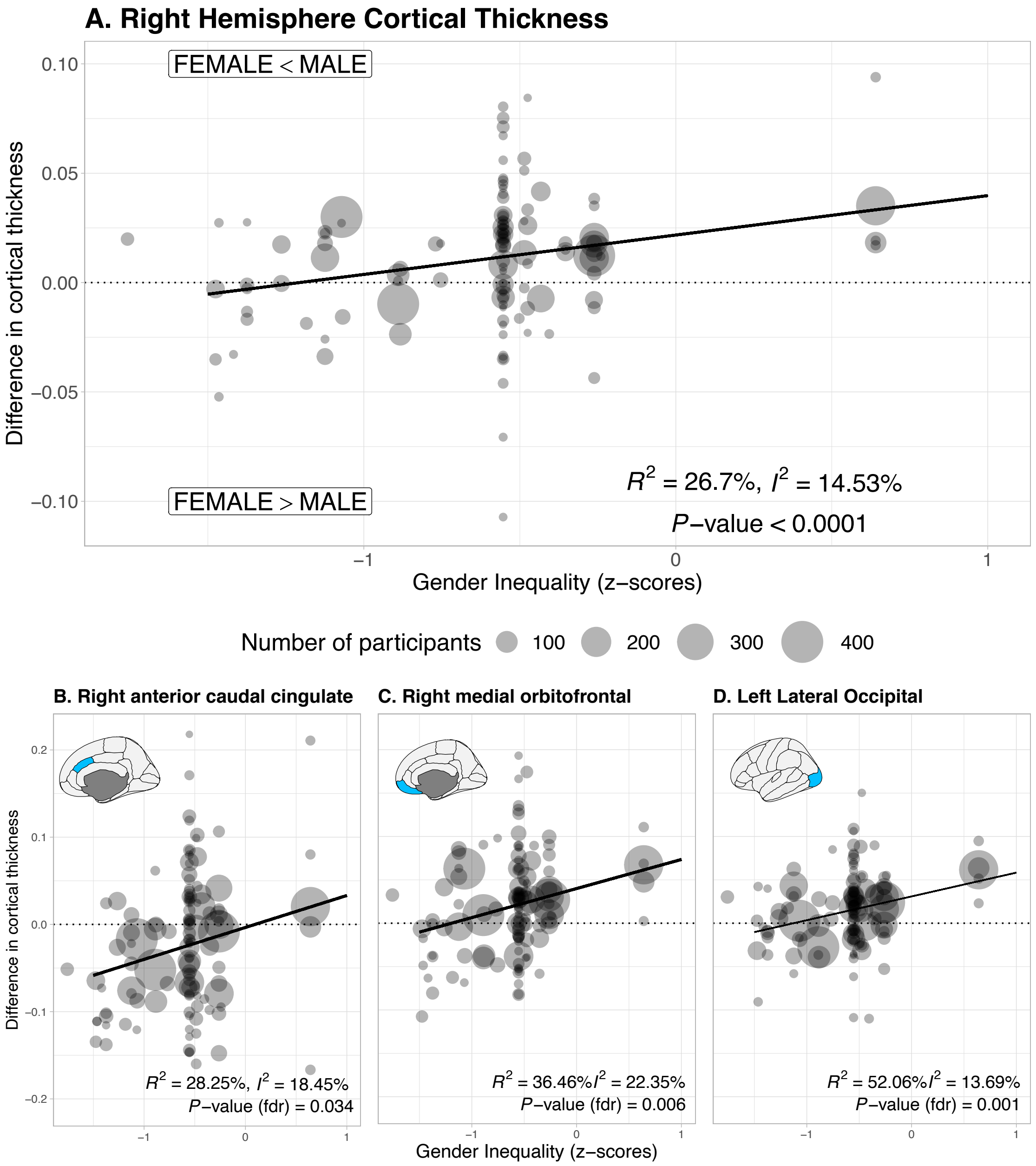

Figure S6. Analyses of cortical thickness only including studies performed on 3T MRI scanners. 119 studies were included, with a total of 6,969 participants. As shown in the figure, results are very consistent with the main analyses.

Differences in cortical thickness in studies with N >= 15

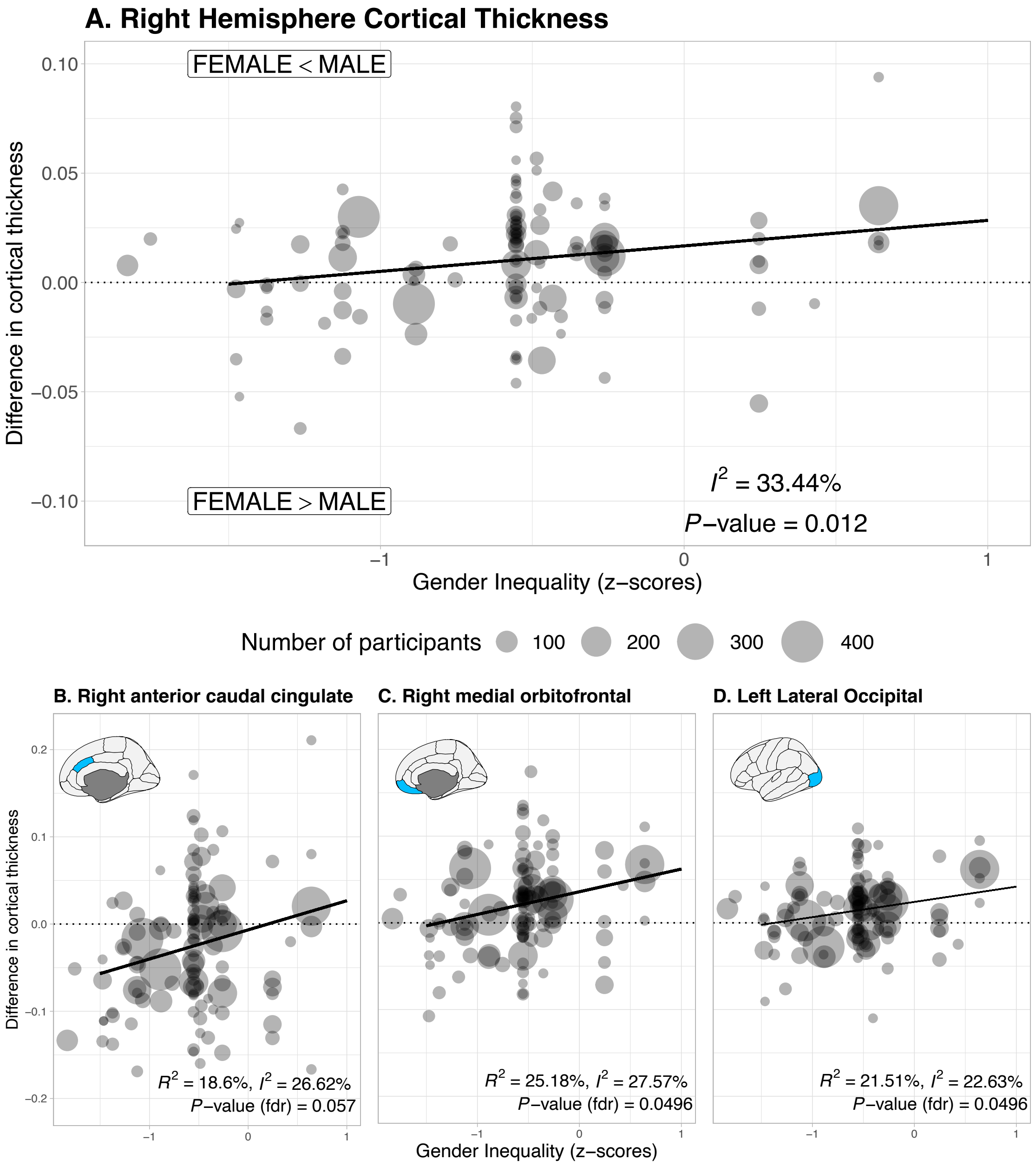

Figure S7. Analyses of cortical thickness only including studies with an N>=15 participants. 113 studies were included, with a total of 7,569 participants. Results were consistent with main analyses, except for the right anterior caudal cingulate being no longer significant after correcting for multiple comparisons (P_{fdr} = 0.057).

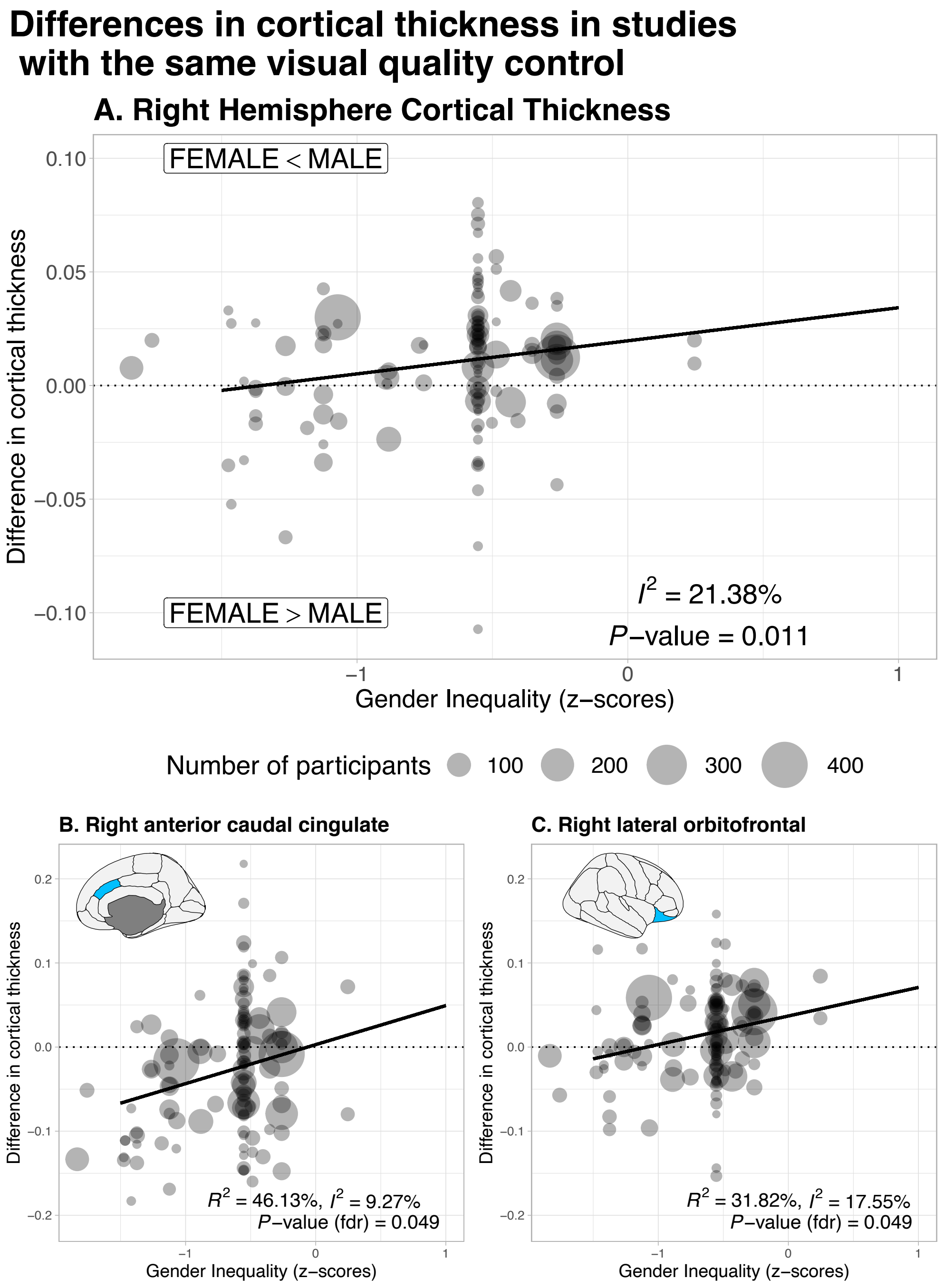

Figure S8. Analyses of cortical thickness only including studies where the visual quality control of the images was performed by the same two reviewers. 116 studies were included, with a total of 6,047 participants. Results in the average difference in the right hemisphere were consistent with the main analyses. Regional results were consistent for the right anterior caudal cingulate, but instead of the right medial orbitofrontal being significant, it was the right lateral orbitofrontal.

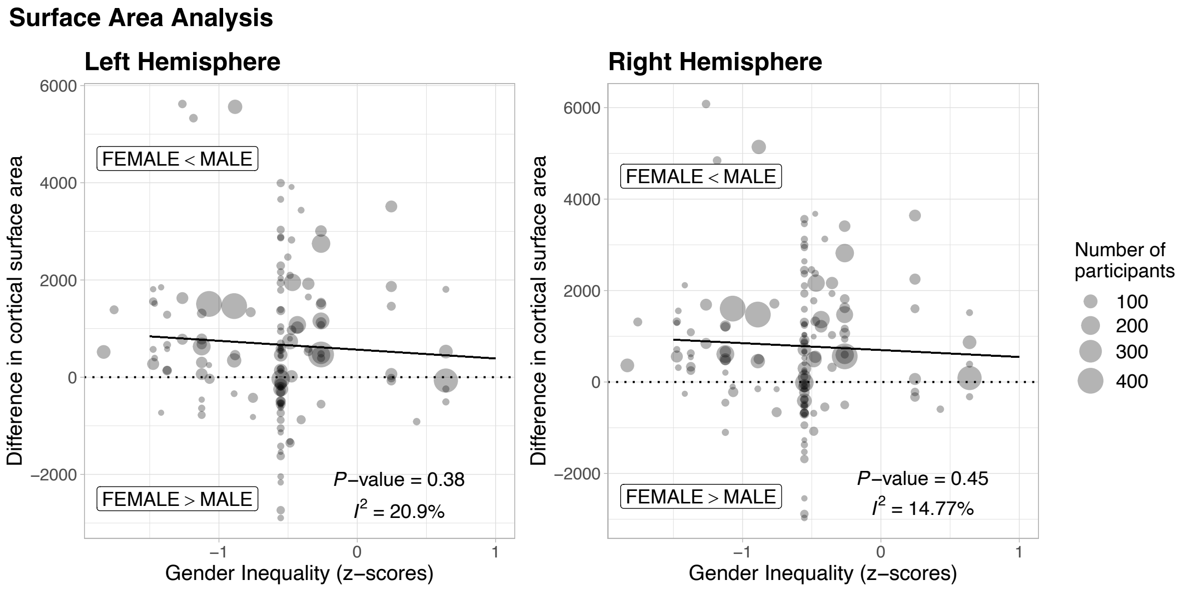

Figure S9. Surface Area Analysis. There were no significant associations between gender inequality and the sex difference in hemispheric surface area, or any of the regions of interest (all $P_{fdr} > 0.05$). Three sites were not included in this analysis since data was not available (Colombia, Cape Town 2 and 3), with a total of 136 samples and 7,822 participants. Analyses correcting for total surface area instead of total intracranial volume, including the full dataset of 139 samples, were not significant for any region of interest either.

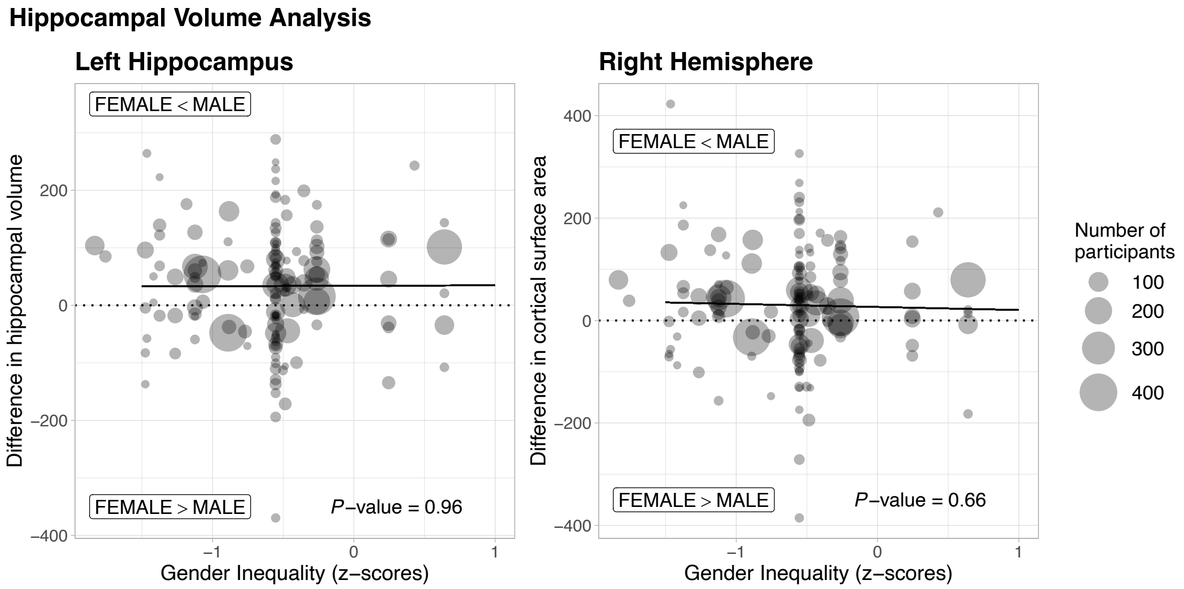

Figure S10. Hippocampal volume analyses. There were no significant associations between gender inequality and the sex difference in hippocampal volumes after controlling for age and total intracranial volume. Data of subcortical structures were available for 137 samples including 7,821 participants.

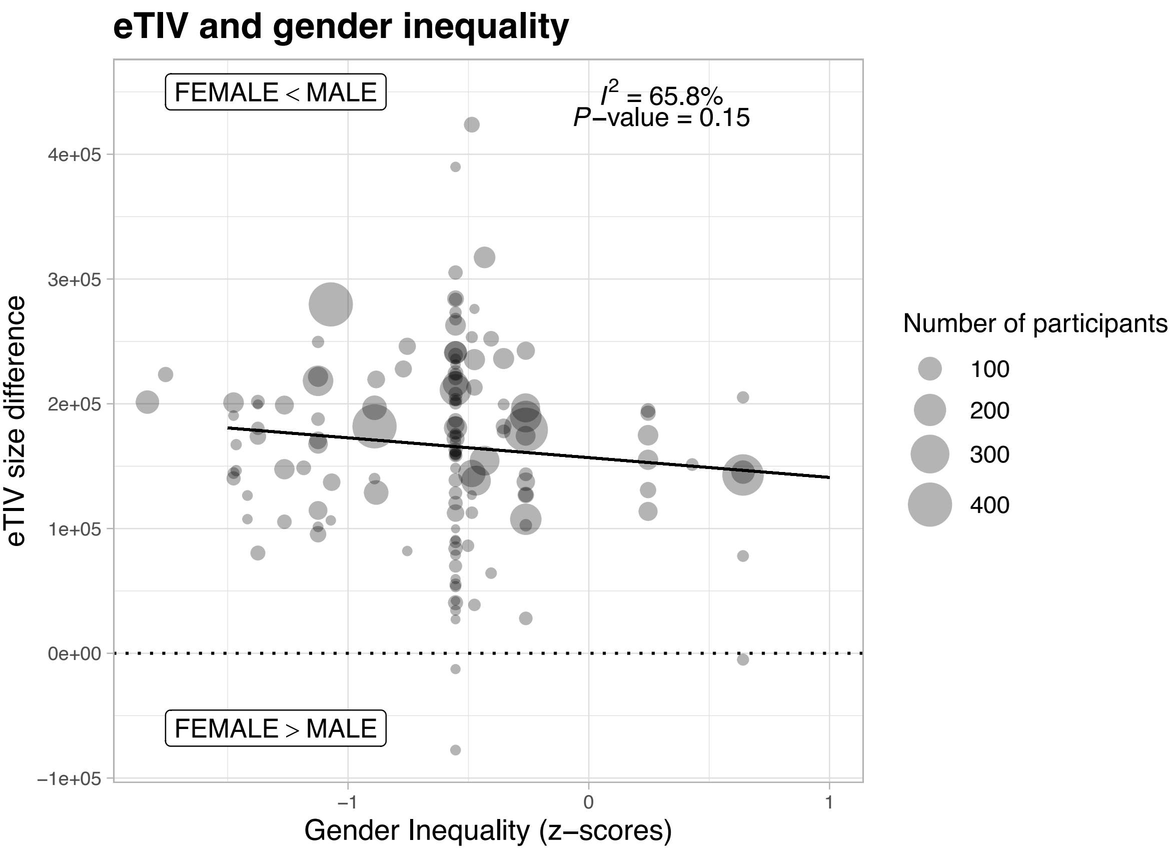

Figure S11. Total intracranial volume and gender inequality. The association between the difference in estimated total intracranial volume (eTIV) between women and men, and gender inequality was not significant (beta -15872, 95%CI -37273 to 5529, P=0.15). As in the area analyses, three sites were not included, with a total of 136 samples and 7822 participants. Controlling for per capita GDP did not change the results (beta -15103, 95%CI -39535 to 9329, P=0.23).

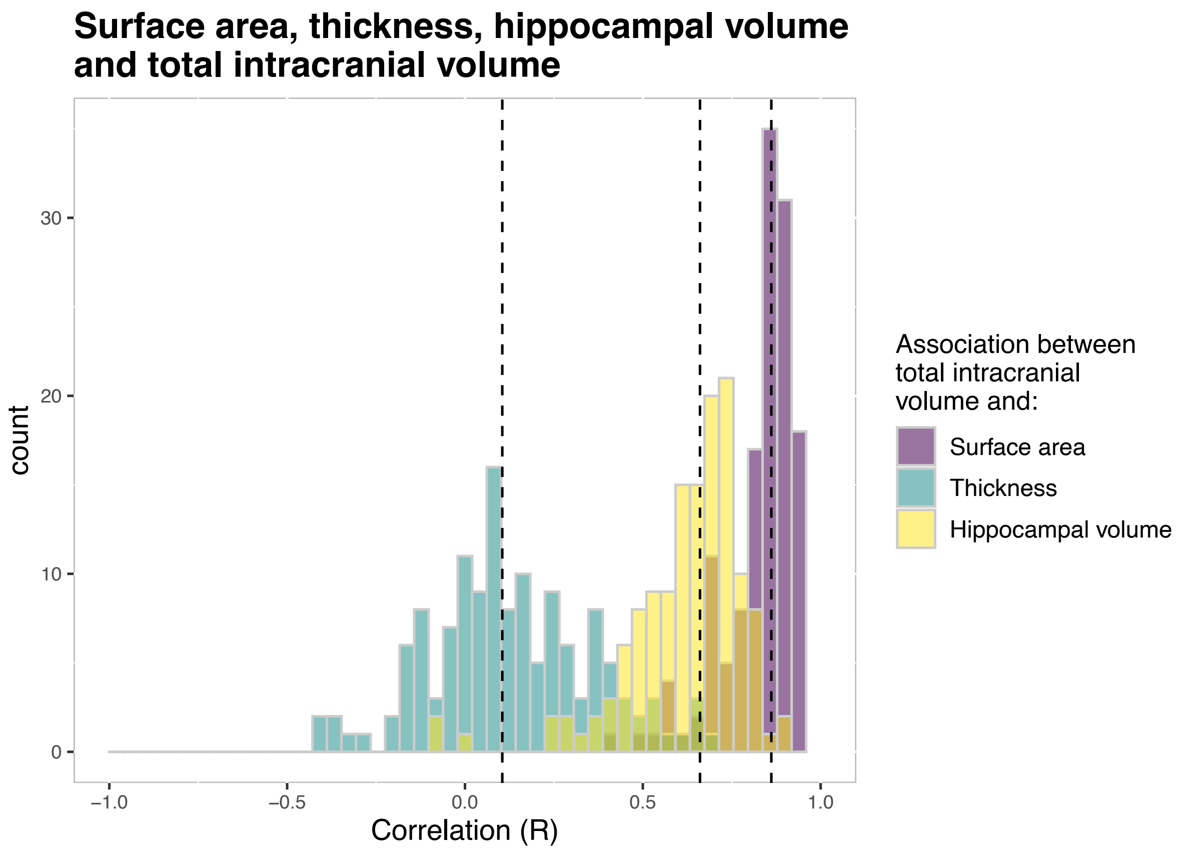

Figure S12. Association between total intracranial volume and whole-brain thickness, surface area and hippocampal volumes. Dashed lines represent median correlation across samples for each morphometric property and intracranial volume. For hippocampal volume the sum of the left and right hippocampi is considered.

PNAS

cVEDA Authors

The following authors were part of the cVEDA:

Pratima Murthy[1]
Amit Chakrabarti[2]
Debasish Basu[3]
B.N. Subodh[3]
Lenin Singh[4]
Roshan Singh[4]
Kartik Kalyanram[5]
Kamakshi Kartik[5]
Kalyanaraman Kumaran[6,7]
Ghattu Krishnaveni[6]
Rebecca Kuriyan[8]
Sunita Simon Kurpad[9]
Gareth J. Barker[10]
Rose D. Bharath[11]
Sylvane Desrivieres[12]
Meera Purushottam[13]
Dimitri P. Orfanos[14]
Eesha Sharma[15]
Matthew Hickman[16]
Jon Heron[17]
Mireille B. Toledano[18]
Nilakshi Vaidya[19]

1. National Institute of Mental Health and Neurosciences (NIMHANS), Bangalore, India.
2. ICMR-Centre on Non-Communicable Diseases, Kolkata, India.
3. Post Graduate Institute of Medical Education and Research (PGIMER), Chandigarh, India.
4. Regional Institute of Medical Sciences, Imphal, Manipur, India.
5. Rishi Valley, Rural Health Centre, India.
6. Epidemiology Research Unit, CSI Holdsworth Memorial Hospital, Mysore, India.
7. MRC Lifecourse Epidemiology Unit, University of Southamtpon, UK.
8. Division of Nutrition, St John's Research Institute, Bangalore, India.
9. Department of Psychiatry & Department of Medical Ethics, St. John's Medical College & Hospital, Bangalore, India.
10. Department of Neuroimaging, Institute of Psychology, Psychiatry & Neuroscience, King's College London, London, UK.
11. Department of Neuroimaging and Interventional Radiology, National Institute of Mental Health and Neurosciences, Bangalore, India.
12. Centre for Population Neuroscience and Precision Medicine, MRC Social, Genetic, Developmental Psychiatry Centre, Institute of Psychology, Psychiatry & Neuroscience, King's College London, London, UK.
13. Molecular Genetics Laboratory, National Institute of Mental Health and Neurosciences, Bangalore, India.
14. NeuroSpin, CEA, Université Paris-Saclay, Paris, France.
15. Department of Child & Adolescent Psychiatry, National Institute of Mental Health and Neurosciences, Bangalore, India.

16. Bristol Medical School, University of Bristol, Bristol UK.
17. Centre for Public Health, Bristol Medical School, University of Bristol, Bristol UK.
18. MRC Centre for Environment and Health, School of Public Health, Imperial College London, UK.
19. Centre for Population Neuroscience and Precision Medicine, Charite Mental Health and Dept. of Psychiatry and Psychotherapy, Charite Universitaetsmedizin Berlin, Germany.

Author contributions:
PM, AC, DB, BNS, LS, RS, KKal, KKar, KKum, GK, RK, SSK, GJB, RDB, SD, MP, DPO, ES, MH, JH, MBT and NV: performed research.
