# Supplementary material for: Country-level gender inequality is associated with structural differences in the brains of women and men
Source: Proc Natl Acad Sci U S A. 2023 May 8;120(20):e2218782120. doi: 10.1073/pnas.2218782120 (PMC10193926; doi:10.1073/pnas.2218782120)
Supplement: Supplementary file 3 — Dataset S02 (DOCX) [file pnas.2218782120.sd02.docx]

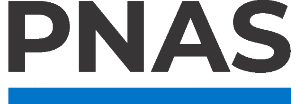


**cVEDA Authors**

The following authors were part of the cVEDA:

Pratima Murthy^1^

Amit Chakrabarti^2^

Debasish Basu^3^

B.N. Subodh^3^

Lenin Singh^4^

Roshan Singh^4^

Kartik Kalyanram^5^

Kamakshi Kartik^5^

Kalyanaraman Kumaran^6,7^

Ghattu Krishnaveni^6^

Rebecca Kuriyan^8^

Sunita Simon Kurpad^9^

Gareth J. Barker^10^

Rose D. Bharath^11^

Sylvane Desrivieres^12^

Meera Purushottam^13^

Dimitri P. Orfanos^14^

Eesha Sharma^15^

Matthew Hickman^16^

Jon Heron^17^

Mireille B. Toledano^18^

Nilakshi Vaidya^19^

1. National Institute of Mental Health and Neurosciences (NIMHANS), Bangalore, India.
2. ICMR-Centre on Non-Communicable Diseases, Kolkata, India.
3. Post Graduate Institute of Medical Education and Research (PGIMER), Chandigarh, India.
4. Regional Institute of Medical Sciences, Imphal, Manipur, India.
5. Rishi Valley, Rural Health Centre, India.
6. Epidemiology Research Unit, CSI Holdsworth Memorial Hospital, Mysore, India.
7. MRC Lifecourse Epidemiology Unit, University of Southamtpon, UK.
8. Division of Nutrition, St John’s Research Institute, Bangalore, India.
9. Department of Psychiatry & Department of Medical Ethics, St. John's Medical College & Hospital, Bangalore, India.
10. Department of Neuroimaging, Institute of Psychology, Psychiatry & Neuroscience, King’s College London, London, UK.
11. Department of Neuroimaging and Interventional Radiology, National Institute of Mental Health and Neurosciences, Bangalore, India.
12. Centre for Population Neuroscience and Precision Medicine, MRC Social, Genetic, Developmental Psychiatry Centre, Institute of Psychology, Psychiatry & Neuroscience, King’s College London, London, UK.
13. Molecular Genetics Laboratory, National Institute of Mental Health and Neurosciences, Bangalore, India.
14. NeuroSpin, CEA, Université Paris-Saclay, Paris, France.
15. Department of Child & Adolescent Psychiatry, National Institute of Mental Health and Neurosciences, Bangalore, India.
16. Bristol Medical School, University of Bristol, Bristol UK.
17. Centre for Public Health, Bristol Medical School, University of Bristol, Bristol UK.
18. MRC Centre for Environment and Health, School of Public Health, Imperial College London, UK.
19. Centre for Population Neuroscience and Precision Medicine, Charite Mental Health and Dept. of Psychiatry and Psychotherapy, Charite Universitaetsmedizin Berlin, Germany.

Author contributions:

PM, AC, DB, BNS, LS, RS, KKal, KKar, KKum, GK, RK, SSK, GJB, RDB, SD, MP, DPO, ES, MH, JH, MBT and NV: performed research.
